# Amplicon sequencing of ice and water phytoplankton and bacterial communities during an extreme winter in a central Canadian great lake

**DOI:** 10.1128/mra.01232-25

**Published:** 2025-12-23

**Authors:** Mark J. Rozmarynowycz, Katelyn M. Brown, J. D. Gantz, Richard E. Lee, Arthur Zastepa, Sue Watson, R. Michael L. McKay

**Affiliations:** 1 Department of Biological Sciences, Bowling Green State University 1888, Bowling Green, Ohio, USA; 2 Great Lakes Institute for Environmental Research, University of Windsor 177440, Windsor, Ontario, Canada; 3 Department of Biology, Miami University 6403, Oxford, Ohio, USA; 4 Canada Centre for Inland Waters, Environment and Climate Change Canada 2085 , Burlington, Ontario, Canada; DOE Joint Genome Institute, Berkeley, California, USA

**Keywords:** Lake Winnipeg, diatom, cyanobacteria, Xanthomonadales, 16S

## Abstract

Ice and water microbial communities from Lake Winnipeg were explored through V4 region 16S rRNA gene sequencing during a pronounced period of cold temperatures over North America’s Great Plains and Great Lakes regions. Diatoms, cyanobacteria, and Xanthomonadales (Gammaproteobacteria) displayed patterns of partitioning into ice fractions.

## ANNOUNCEMENT

Pronounced cold temperatures dominated weather over North America’s Great Plains and Great Lakes regions in winter 2013–2014 (1, 2). Freshwater ice serves as a habitat for phytoplankton and their associated microbial communities, with distinct partitioning evident between ice and water (3, 4). Here, we use 16S rRNA gene sequencing of ice and water communities in Lake Winnipeg, Manitoba, Canada, to identify how microbes differ between these fractions.

Environment and Climate Change Canada hydrographic stations in Lake Winnipeg were accessed by snowmobile during February 2014 (Table 1). Surface water was collected from holes augered using a SPIRE hand auger (76 mm diameter; U.S. Ice Drilling Program, Madison, WI) (4). Ice cores were measured, rinsed with sterile water, and placed in new sealable plastic bags (S.C. Johnson & Son, Racine, WI). Water and ice cores were transported on ice to the laboratory for processing. Biomass from water and ice melted in a cooler overnight at room temperature was separately concentrated on Sterivex (0.22 µm; EMD Millipore, Billerica, MA), flash-frozen in liquid nitrogen, and stored at −80°C. DNA was extracted with a PowerWater Sterivex DNA Isolation Kit (MO BIO Laboratories, Carlsbad, CA). 16S rRNA gene sequencing was completed at Joint Genome Institute (Berkeley, CA) using an Illumina MiSeq (2 × 250 bp reads; Illumina, San Diego, CA (5, 6). A 5 PRIME HotMasterMix Amplification Kit (5 PRIME, Montreal, QC) was used with primers 515F (5′-CACGGTCGKCGGCGCCATT-3′) and modified 806R (6) for V4 region amplification. Water and ice melt were preserved with 0.5% paraformaldehyde for flow cytometry at the J.J. MacIsaac Facility for Aquatic Cytometry (Bigelow Laboratory for Ocean Sciences, East Boothbay, Maine) with a Beckman Coulter Legacy MoFlo instrument (7).

**TABLE 1:** Sampling metadata and accession numbers of sequenced samples from Lake Winnipeg in 2014^*a*^.

Site ID	Location	Date (2014)	Water sampling depth (m)	NO_3_ (mg L^−1^)	NH_3_ (mg L^−1^)	SRP (µg L^−1^)	Cl (mg L^−1^)	SO_4_ (mg L^−1^)	No. phototrophic eukaryotes (< 20 µm, cells mL^−1^)	Total phytoplankton (cells mL^−1^)	Total reads	Filtered reads	Accession no.
W2	53.263, −99.024	22-Feb	0.5	0.08	0.04	6.6	16.4	67.5	389	872	221,274	175,199	SRR35572707
W2 – Ice	53.263, −99.024	22-Feb	–	0.02	0.052	10	1.2	1.7	112	207	223,248	181,536	SRR35572698
W9	51.022, −96.584	27-Feb	0.5	0.08	0.032	15.2	1.6	3.7	2,381	3,340	262,734	205,602	SRR35572705
W9 – Ice	51.022, −96.584	27-Feb	–	0.02	0.028	4.2	0.3	0.5	112	376	269,569	216,023	SRR35572704
W12	50.516, −96.834	27-Feb	0.5	0.18	0.031	106.3	12.9	87	2,899	7,276	128,548	102,029	SRR35572708
W25S	53.209, −98.901	23-Feb	0.5	0.1	0.051	5.1	16.2	69.5	405	901	336,329	268,813	SRR35572703
W25S – Ice A	53.209, −98.901	23-Feb	–	n.d.	n.d.	n.d.	n.d.	n.d.	12	806	181,254	145,230	SRR35572702
W25S – Ice B	53.209, −98.901	23-Feb									224,606	179,316	SRR35572701
W25S – Ice C	53.209, −98.901	23-Feb									271,095	219,645	SRR35572700
W28	53.213, −99.233	23-Feb	0.5	0.09	0.045	6.9	17.3	68.7	827	1,802	247,333	197,392	SRR35572699
W41	52.821, −98.497	24-Feb	0.5	0.09	0.041	22.9	21.8	39.3	244	2,307	281,076	225,755	SRR35572697
W41 – Ice A	52.821, −98.497	24-Feb	–	0.02	0.033	5.1	0.9	1.5	12	318	215,030	172,654	SRR35572706
W41 – Ice B	52.821, −98.497	24-Feb	–	0.01	0.043	14.3	4.7	9.1	21	1,827	255,245	202,131	SRR35572696

^*a*^ Nutrients (NO_3_ [nitrate], NH_3_ [ammonia], SRP [soluble reactive phosphorus], Cl [chloride], SO_4_ [sulfate]) were measured at the National Center for Water Quality Research at Heidelberg University (Tiffin, OH) using standard methods (8) and are shown as dissolved (<0.22 µm; Sterivex filtrate) concentrations. n.d., no data. Dashes in water sampling depth indicate no data due to the sample being ice.

Adapters were filtered, unpaired reads discarded, sequences trimmed to 165 bp, and assembled using FLASH (9). Sequences were demultiplexed and quality filtered: sequences were trimmed (sliding window=10 bp), required to display a mean quality score of 30 or more, and to contain less than 5 Ns or 10 bases with a quality score less than 15. With MacQIIME 1.9.1 (10), operational taxonomic units (OTUs) were clustered at 97% similarity (uclust)(11). OTUs represented three times or fewer were filtered, and a representative set of sequences was created for the most abundant sequence in each cluster. Naive Bayes classifier software (12) was used to assign taxonomy based on the Greengenes database v.12_10 (13, 14). Sequences were aligned to the Greengenes Core reference alignment (15) using PyNAST (16), and gaps were filtered. Default parameters were used unless otherwise noted.

**Fig 1:**
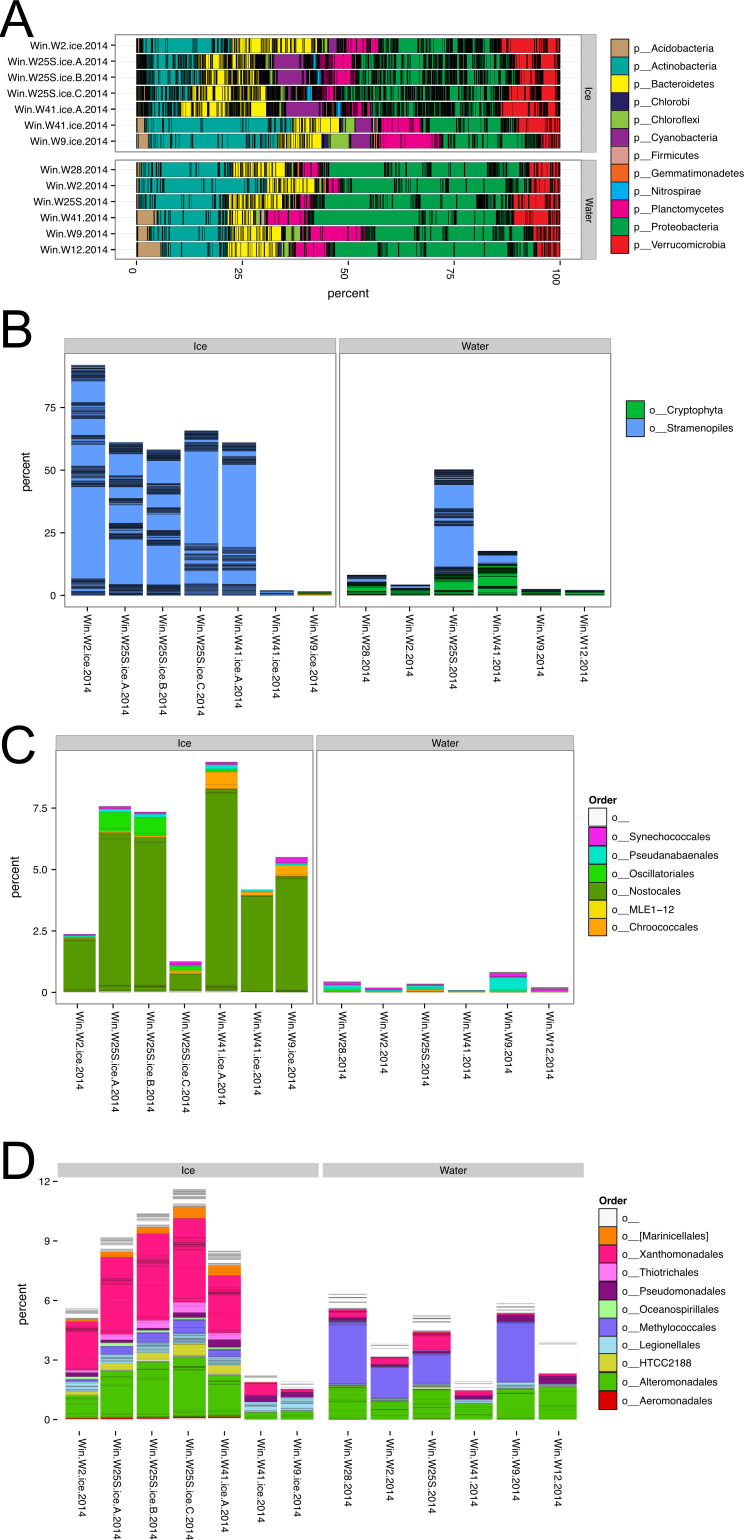
Community composition of Lake Winnipeg ice and water for (**A**) the complete bacterial community at the phylum level, (**B**) photosynthetic microeukaryotes at the order level, (**C**) cyanobacteria at the order level, and (**D**) Gammaproteobacteria at the order level.

Ice and water communities were similar, with Proteobacteria contributing a large portion of the overall community, but taxa like Cyanobacteria were only observed in the ice samples (Fig. 1A). Chloroplast 16S rDNA revealed that Stramenopiles were in greatest relative abundance in ice and water (Fig. 1B). Cyanobacteria preferentially partitioned to ice (Fig. 1C), and a similar pattern was seen for Xanthomonadales (Gammaproteobacteria; Fig. 1D), consistent with their role in biological ice nucleation (17) and an elevated mean temperature of crystallization measured for ice melt water of −6.87°C (18).

## Data Availability

The raw reads from this amplicon sequencing project have been deposited in NCBI Sequence Read Archive under the BioProject accession PRJNA1333487.
